# Patients’ perspectives on providing a stool sample to their GP: a qualitative study

**DOI:** 10.3399/bjgp14X682261

**Published:** 2014-10-27

**Authors:** Donna M Lecky, Meredith KD Hawking, Cliodna AM McNulty

**Affiliations:** Public Health England Primary Care Unit, Gloucester.; Public Health England Primary Care Unit, Gloucester.; Public Health England Primary Care Unit, Gloucester.

**Keywords:** information leaflet, opinion, patient interview, primary care, qualitative, stool collection

## Abstract

**Background:**

Stool specimen collection is challenging and informal feedback has indicated that participants find the process difficult. Increasing stool specimen returns would improve the investigation of outbreaks of diarrhoeal and food-borne disease.

**Aim:**

To explore the barriers to stool sample collection and specimen return to ascertain which factors may help to improve the process.

**Design and setting:**

Qualitative patient interview study in Gloucester, UK.

**Method:**

A two-stage purposive sampling process was used to identify patients who had either previous experience or no experience of collecting a stool sample. The interview schedule, based on the theory of planned behaviour, was used to facilitate interviews with 26 patients. Interview transcripts were analysed using a modified framework analysis.

**Results:**

Barriers to collection included embarrassment, fear of results, concerns around hygiene and contamination, discretion and privacy, and lack of information. Personal gain was identified as the main incentive to collecting and returning a stool sample. The need for an information leaflet on stool collection was emphasised by most patients.

**Conclusions:**

GPs could make a number of small changes that could make a big difference for patients and potentially increase stool sample return. If they, rather than receptionists, distributed collection kits it may be easier for patients to ask any questions they had regarding collection. In addition, the provision of a stool-collection information leaflet could increase patients’ confidence regarding collecting the sample, and providing drop-off boxes for specimens could help prevent patients’ embarrassment regarding handing their stool over to a receptionist.

## INTRODUCTION

Collecting a stool specimen can be a definitive step in determining the diagnosis and appropriate treatment for suspected infectious diarrhoea and other gastrointestinal disease.^1^ Stool culture results also form the basis of ongoing surveillance of infectious diarrhoea in the community.^2^ Stool specimens may also be required for non-microbiological testing, for example faecal occult blood testing (FOBt), an early detection method for colorectal cancer.^3^ Despite the fact that early FOBt screening has been shown to reduce mortality by 16%,^3^ compliance is rarely >60%;^4^ reasons given for the lack of patient compliance with stool collection for FOBt include embarrassment, concerns about screening results, and inconsistent or inadequate support from friends and family.^5^

To the best of the authors' knowledge, no research has been published examining the patient perspective of collecting stool samples for microbiological testing. Due to the lack of current research in the area, this study aimed to explore, through patient interviews, the barriers to stool specimen collection and return, as well as factors that may help to improve the process. It was hoped that the results would inform the improvement of stool collection instructions for patients participating in a stool surveillance study and improve national guidance for GPs and patients on the investigation of suspected infectious intestinal disease.

**Figure 1.**
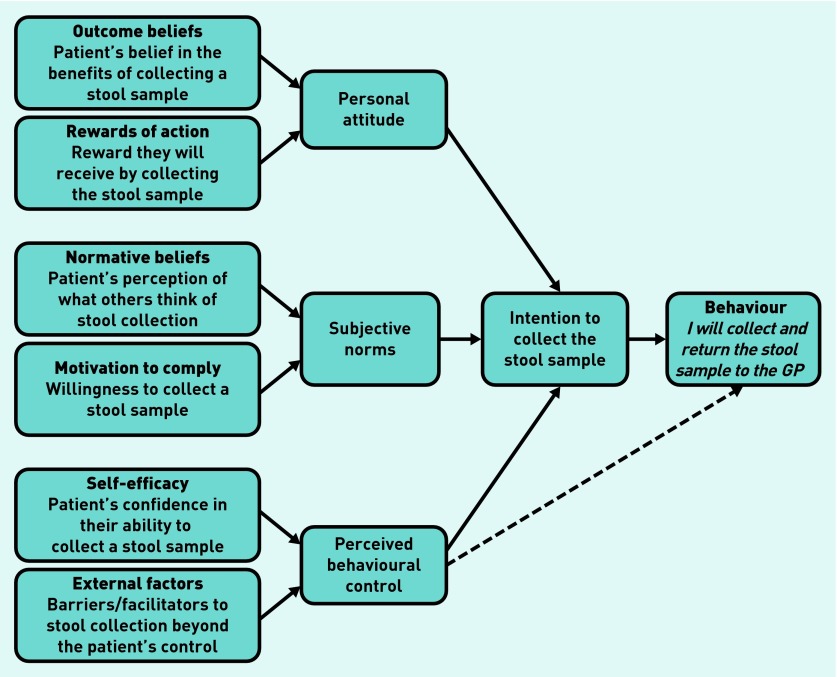
***Theory of planned behaviour intention to collect a stool specimen for microbiological testing.***

Current GP guidance focuses on the treatment of diarrhoea^6^ and not the collection of the stool sample itself. The theory of planned behaviour^7^ has been used to develop the interview schedule to identify patients’ attitudes, subjective norms, and perceived behavioural control to submitting a stool sample in order to determine their intentions to act. The model suggests that individuals are more likely to intend to perform a behaviour (returning a stool sample) if they have a positive attitude towards it, perceive social pressure from others to perform the behaviour (subjective norms), and perceive that the performance of the behaviour is within their control (perceived behavioural control)^8^ (Figure 1).

## METHOD

### Participant selection and recruitment

A two-stage purposive sampling process was used to identify patients who had either previous experience (Group 1) or no previous experience (Group 2) of collecting a stool sample. Those included were patients aged >18 years, who had submitted a stool (Group 1) or blood sample (Group 2), as requested by a GP, to the microbiology department of Gloucestershire Royal Hospital in the 3 months leading up to the recruitment phase. Women who were pregnant or patients who were terminally ill were excluded.

Potential participants were stratified by ethnic origin, age, sex, and stool consistency (Group 1 only). White patients were selected randomly from each stratified list. All patients of non-white ethnicity were invited to take part due to their low numbers. The researchers aimed to recruit up to five participants from each stratum.

**How this fits in**

Stool specimen collection is needed to inform the management of many gastrointestinal diseases and infections, but returns by patients are generally <60%. Through interviews based on the theory of planned behaviour with patients who had and had not submitted stool specimens, it was found that personal attitudes, subjective norms, and perceived behavioural controls all influenced specimen return. Patients perceived that handling stools was dirty and embarrassing, and it was found that a lack of information about why patients were collecting the stool, how to do it, receptionist involvement, privacy during returning specimens, and fear of results were all barriers to collection. Stool specimen returns may be increased through greater explanation about the reason for collection by the GP, providing the patient with plastic gloves (or telling them where to get them), and giving the patient Public Health England’s patient information leaflet on stool collection, which includes diagrams and opaque bags for return.

Invitation letters containing study information from the local microbiologist and Public Health England Primary Care Unit (PCU) were sent to all selected patients. Letters describing the study were also sent to each participant’s GP practice. Willing participants provided written informed consent. Telephone calls were used to organise interview dates and venues. Patients were reimbursed for their time with a £10 voucher.

### Interview sessions

Participants selected whether they preferred to be interviewed in their own home or a hospital office. Where possible, interviewers were matched in both ethnicity and sex to the participants.

Interviewers used a flexible interview schedule that had been developed by the investigators based on the theory of planned behaviour and comprised a mixture of closed and open-ended questions (Appendix 1). Visual aids in the form of a standard stool collection kit available in England and patient information leaflets were used to facilitate interviews. Each interview session lasted approximately 45 minutes.

### Analysis

All interviews were recorded, with permission, and transcribed verbatim. Transcripts were analysed using a modified framework analysis. Transcripts were first read through for accuracy and to gain knowledge of the data. Two researchers independently coded categories and themes: the lead researcher coded all transcripts and a second researcher coded 10% of the transcripts to ensure coding consistency. The researchers then agreed categories and themes; discrepancies were resolved through discussion and referral to original transcripts until agreement was reached.

Themes were revised iteratively as the fieldwork and analysis progressed.^9^ Use of NVivo software (version 10) facilitated the organisation of the data. The one sheet of paper (OSOP) method was used to clarify findings within, and between, themes.^10^

## RESULTS

**Figure 2.**
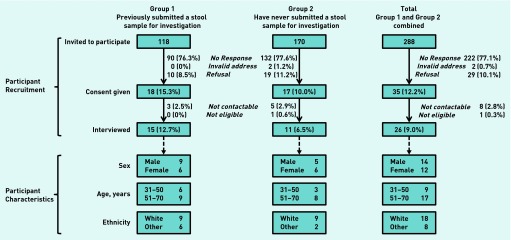
***Participant recruitment and characteristics.***

Between March 2012 and June 2013, a total of 288 patients (Group 1, *n* = 118; Group 2, *n* = 170) were invited to participate. Thirty five (12.2%) agreed to do so; however, only 26 (9.0%) patients were interviewed due to patient drop out (Figure 2).

Stool collection methods previously used by Group 1 varied between participants but stool consistency had no effect on the collection method. Participants felt that the collection of a stool sample would be easier next time they were asked to do so. Forty per cent of Group 1 participants found it harder than they had expected due to lack of knowledge or instructions on how to collect the sample, the consistency of the stool, or the fact that it was messy. Group 2 participants’ main concern on stool collection was not knowing how to collect the specimen.

Overall six major themes emerged from the interviews:

- barriers to collection;
- incentives to collection;
- information and support;
- collection management;
- relationships; and
- information and support.

These themes were further examined using the theory of planned behaviour model as outlined below.

### Personal attitudes: outcome beliefs

#### Lack of information

Participants with previous experience of stool collection highlighted a lack of information from the GP on how to collect a stool sample:

‘But I didn’t know what to do. I thought “how do you, how do you catch it here,” I thought, “without it ending up in the water?” So I thought if I pee or poo on some cardboard then take a little bit off and then that’s it in there?’

(Group 1, participant 5)

‘I think it might have been more helpful as to what they were, you know, what to include in, in the sample, then you know not just ordinary poo but the mucus and you know what, what they really wanted, so I think that would be more helpful.’

(Group 1, participant 1)

#### Fear of results

Fear of the possibility of receiving bad results was also a frequent response:

‘People, I mean I think people, um, don’t want to know the worst, and I’d rather live with what I don’t know than try and live with what I do know type of thing. I think I can be a bit like that sometimes.’

(Group 2, participant 4)

‘Sometimes not wanting to know the results. They know something is wrong but they don’t… sometimes some people can’t deal with it.’

(Group 2, participant 11)

Such a response was particularly the case when patients wrongly assumed the request for a stool sample was linked to colorectal cancer screening.

### Personal attitudes: rewards of action

#### Personal gain

The main driver to returning the sample appeared to be personal benefit:

‘I think if it’s to my benefit, I would do it.’

(Group 1, participant 3)

‘And somehow or other you have to create a shift in responsibility, that is, the patient takes responsibility for their own health and they’re having a stool sample tested because it’s clinically necessary.’

(Group 2, participant 8)

If the patient understood why they were being asked for the sample and when they should expect to get the results, they were happier to provide the sample:

*‘They* [GPs] *have obviously got to emphasise what the results are going to show and whether that will have a positive or negative effect on your own personal health so therefore it has got to be stressed why they are doing it.’*

(Group 2, participant 10)

### Subjective norms: normative beliefs

#### Embarrassment

The main barriers to stool collection included the embarrassment of other people knowing that they were carrying their stool in a container:

It’s embarrassing, it’s just a subject that is, isn’t it? I think what I found embarrassing about it was that it was a clear jar so when you’re handing it over and it’s in clear polythene as well … I found that very embarrassing. You know, “here’s my sample”.’

(Group 1, participant 1)

In addition, participants were embarrassed about the actual process of collecting the sample as you are ‘taught not to handle poo’; they felt that this may be a main reason why some people lack the motivation to comply:

*‘We have all been taught not to handle and deal with poo, and being embarrassed that you have had to do it yourself …* [It] *might be something that they don’t want to do.’*

(Group 2, participant 10)

### Subjective norms: motivation to comply

#### Patient–GP relationships

Participants reported an extremely trusting relationship between themselves and their GP. All participants said they were happy for a GP to ask them for a stool sample without questioning why:

‘You don’t question your doctor. You know it’s in your own interest really.’

(Group 1, participant 12)

Participants also viewed their GP as the main source of information on stool collection:

*‘How did I find discussing it? Um, well we didn’t really. Um, we didn’t at all. I mean, he told me what it was for um, and then* […] *I went to reception and asked for a stool sample bottle and she gave it to me and that was it. There wasn’t really any discussion about it.’*

(Group 1, participant 1)

‘There wasn’t much discussion. It was just “give me a stool sample and go to the um, receptionist, err, to get it” and that was it.’

(Group 1, participant 4)

#### The family relationship

This plays an important part in, not only seeking medical advice, but also returning stool samples. Although, in many cases, participants were self-motivating in visiting the GP, most of them, particularly females, discussed the sample request openly with their family:

‘Well I’d discuss it with my sister at the weekend because she and her husband have, um, both had to do samples recently. So um, yes, it’s not something we’d discuss but it’s something I might mention in passing ... we didn’t really go into details of how it works and what it is.’

(Group 2, participant 4)

Men more often said that they would only discuss it with their partner/wife:

‘Well my wife would know because I would tell her but she wouldn’t have a problem as long as there was a reason for me having to do it.’

(Group 2, participant 10)

Male participants were the only ones who stated that they would not discuss this with their family.

#### Stool collection kits

Participants expected more from kits than what is currently provided by GP practices:

‘I think that is a pathetic little kit. It’s insufficient, it’s … patients need as much information as possible and possibly you need to bore them with it, but a lot of what is said to a patient in a consulting room that’s forgotten when they leave. So all the instructions about how to go ahead and collect a stool sample needs to be in this.’

(Group 2, participant 8)

Expectations included instructions, a collection device such as a spoon or spatula, a collection pot, a coloured/paper bag in which to return the sample, and a pair of gloves to facilitate collection. As Group 1, participant 3, suggested:

‘It could include a pair of surgical gloves, maybe help with that might encourage people to do it.’

Participants in Group 2 suggested that the lack of these materials may be a reason why some people do not return their sample.

Most participants from Group 1 first saw the stool collection kit when collecting it from the receptionist after the GP consultation. At that point, participants in Group 1 reported not feeling comfortable asking the receptionist questions but said it was too late to ask their GP.

Most participants reported that they would not be happy for the receptionist to ask them to collect a stool sample, mainly because they were not professionally trained. Those who were happy for the receptionist to ask for a sample felt it was because the GP had requested it.

### Perceived behavioural control: self-efficacy

#### Information leaflets

Although only two participants received an information leaflet, the majority said they would have expected or liked to have been offered one. Participants wanted information on:

- why the sample was required;
- how much sample was required;
- how to collect the sample;
- where they should return it; and
- when they should expect to get the results.

All participants stated that any information leaflet should have images, large font and wording that would be easily understood by the general public. Participants also stated that the leaflets should:

- include a step-by-step guide using images or diagrams;
- have clear, concise information in large font;
- be in simple language that is easy to understand;
- be in colour;
- have a professional appearance;
- be available in different languages;
- be available on the internet to save the surgery or NHS printing costs;
- provide information on how to collect the stool sample, why collecting the sample is important, when you should expect to receive the results, how to dispose of unwanted material, and emphasise the importance of washing hands; and
- state where to hold the sample overnight (preferably not in the fridge).

### Perceived behavioural control: external factors

#### Hygiene concerns

Getting their hands dirty, handling faeces, or disposing of the sample, and putting their hands near the toilet pan were common issues raised by participants:

‘Well, I didn’t particularly want to get my hands dirty. Um, it’s something you can’t, you’re kind of brought up to, you know, that’s dirty and you don’t wanna touch it or ...’

(Group 1, participant 11)

‘I don’t like the idea of having to put your hand into the toilet as well when you’ve already been to the toilet.’

(Group 1, participant 17)

Six responders expressed concerns around potential contamination of the sample during collection or of the sample leaking and contaminating other things:

‘… and as long as it wasn’t getting contaminated or anything like that then um, …. Equally it could … ah … the sort of thing, if it got damaged, it wouldn’t be very nice and also ah … the risk of, I don’t know, contamination maybe.’

(Group 1, participant 12)

Concern over how to dispose of collection devices was mentioned by participants who had no previous experience of stool collection:

‘And then something to dispose the receptacle that you’ve caught it in, because you’re not going to want to put that in the bin and you’re not going to want to put it down the loo either, so what are you supposed to do with it?’

(Group 2, participant 8)

#### Discretion and privacy

Discretion and privacy were extremely important to patients regarding the request and return of a stool sample; many saw the receptionist as a barrier to this as they were not a medical professional:

‘Um, if a receptionist asked me. Yeah I mean it’s not the nicest place the reception is it? You got a bit more of a public presence out there. Ahhh, and yeah you don’t wanna be discussing. So if ah … I mean if it was done discreetly — and I’m sure they’d try to — but ah … receptions are always a busy little area so it would not be an ideal place. It wouldn’t be my first choice anyway, if it was like a little room or a doctor’s own room then you got more privacy really.’

(Group 1, participant 12)

‘Well, as long as there was a certain amount of privacy involved, yes. You don’t want the whole waiting room listening!’

(Group 2, participant 8)

Responders indicated that, as the reception area lacked privacy, they would prefer to return the sample in a drop box or post it to the surgery, especially as the stool collection container was transparent and no opaque bag or cover was provided to return the sample. Ease of returning the sample was strongly linked with discretion and privacy:

‘Oh that might be easier because then you can just go in, pop it in the box and then leave then without having to queue, you hand it over the reception desk, just drop it off it might be easier.’

(Group 1, participant 4)

‘It was actually a lot easier putting it into a box than giving it to a person.’

(Group 1, participant 11)

## DISCUSSION

### Summary

The present study used the theory of planned behaviour to develop an interview schedule to help identify patients’ attitudes towards collecting and returning a stool sample to their GP for microbiological examination. It is evident from this study that patients’ personal attitudes have a major influence on stool collection; patients do not view the request for a stool sample as routine and, therefore, fear the request and future results. In this instance they do not perceive any *reward* for their action.

The embarrassment of other people, including receptionists, knowing that patients had collected a stool sample and the taboo associated with the ‘dirtiness’ of human faeces were key subjective norms. Participants felt that this may be a key reason why some people lack the motivation to comply.

The greatest barrier to collection was perceived as being a lack of confidence or a lack of instructions on how to collect the sample, along with the undesired involvement of the receptionist in the process.

### Strengths and limitations

To the best of the authors' knowledge, this is the first study examining the patient perspective of collecting and submitting a stool sample for routine microbiological examination. The findings are of particular importance because all participants were primary care patients and their responses reflect real issues about stool collection in this setting; previously, much research in this area has been with participants involved in colorectal cancer screening.^4^,^5^

Although 27% of participants were non-white, the Asian community were under-represented and therefore the study could not identify cultural barriers to stool collection for this group.

Although the majority of participants in this study were aged 31–71 years, no differences between age groups were identified and so the authors believe that these findings will also be applicable to younger patients.

### Comparison with existing literature

In line with the findings presented here, a survey of South Asian women in England found that the most important factors affecting FOBt response related to the difficulty of collecting the stool specimen;^11^ other research has also suggested that perceived provider attitudes play an extremely important role in how comfortable patients feel in returning a stool sample.^12^–^14^

Other literature has also suggested that patient health outcomes can be improved with good GP–patient communication.^15^ In 2012, in almost one-quarter of calls to The Patients Association’s helpline, patients said their GP reacted ‘very poorly’ and refused to talk about their concerns.^16^ An open dialogue between patient and doctor may go some way to removing the taboo associated with handling human faeces and the advice given could alleviate patient concern of sample contamination.^17^ When discussing colorectal cancer screening Hynman *et al,*^18^ suggested that, to increase screening compliance, the potential illness should be explained to the patient to allay fears of hospitalisation and treatment, and that benefits should be explained to overcome reservations.

In the study reported here, it was found that most patients were asked to collect the stool collection kit from the receptionist. However, patients did not view the receptionist as a health professional and, therefore, did not feel comfortable asking questions about stool collection. Although the focus of this study was not on the patient–receptionist relationship, previous studies found that receptionists were perceived as rude, impersonal, insensitive or officious,^19^ which may further explain the reasons behind patients’ perceived embarrassment in returning the sample to the receptionist.

The King’s Fund recently reported that practices performing well on delivering a good experience for their patients also perform well on measures of clinical quality.^20^ In a recent English survey, 89% of GP responders (*n* = 477)^21^ reported giving verbal advice to patients on how to collect a stool specimen although only 2% gave written instructions;^22^ this is despite the fact that it has been shown that screening compliance is significantly improved when patients have an information leaflet.^23^

### Implications for practice

The findings of this study suggest that patients trust the judgement of their GP; as such, there are a number of small changes that GPs could implement that could make a large difference to patients and potentially increase their stool sample returns.

#### Improving personal attitudes

GPs, rather than receptionists, could give patients the stool collection kits; this would likely increase patient confidence in stool collection and allow patients to ask questions about the process. Explaining why the sample is required may remove the fear associated with being asked to submit a non-routine sample request, as well as highlighting the personal benefit to the patients of returning the sample.

#### Removing negative subjective norms

GPs discussing stool collection with their patients may also help to remove the taboo associated with stool collection. The provision of drop-off boxes for sample return may also help remove the embarrassment associated with returning the sample to the receptionist in front of other patients.

#### Increasing perceived behavioural control

The provision of gloves, or advice about where gloves could be obtained, may also help to remove the perceived ‘dirtiness’ that is associated with stool collection and, in turn, increase sample return. In addition, the provision of an opaque bag in which to return the sample, for example, would meet patients’ needs for privacy and discretion.

This research also found that participants wanted — and expected to receive — a stool collection information leaflet. The authors have used the patients’ suggestions to develop a new stool collection information leaflet for those participating in a stool surveillance study (Appendix 2); this will be modified for use with GPs and patients undergoing investigation of suspected infectious intestinal diseases. The modified leaflet will be made available alongside the management of diarrhoea guidance on Public Health England’s website in the next few months, and can also be requested directly from the lead author, but should also be promoted via GP computer systems, NHS information sources, laboratories, and through other professional bodies.

## Figures and Tables

**Appendix 2.**
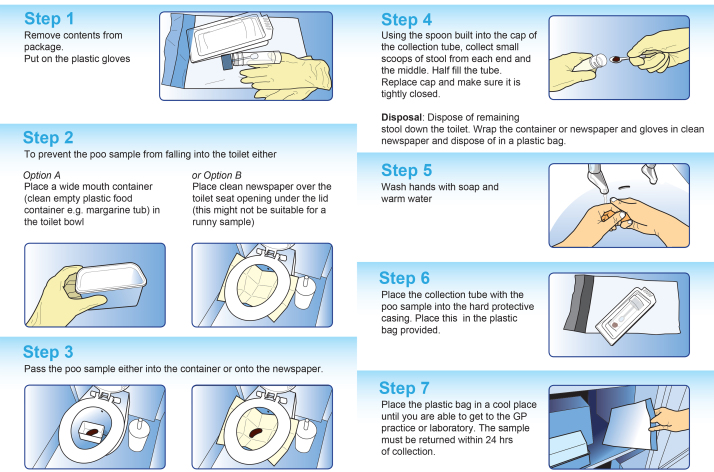
***Stool collection information leaflet for patients.***

## Appendix

**Appendix 1.** Interview schedule

**Participant questions^a^**
**Part 1 – your personal experiences**
Can you remember when you /*Have you ever submitted* your last stool sample for investigation? Could you explain how you collected your most recent stool sample? (stool only) Could you tell me why you recently returned a stool specimen? (stool only) Did you expect to be asked to submit a specimen considering the reason you visited the GP? (stool only) Did anyone encourage you to see your doctor? (stool only) Who asked you to provide a stool sample? (stool only) What explanation, if any, did they give for requesting the sample? Did they give you any information on how to collect the stool sample? How did you find discussing stool collection? How do/did/*would* you feel about being asked to collect a stool sample? When you were given the stool collection kit, did you / *If your GP requested a stool specimen, do you think you would* worry about how to collect the stool sample? When you collected the sample did you find it easier or harder than you had expected? Why? (stool only)
**Part 2 – your general opinion on collecting and submitting a stool sample**
How would you feel if you were asked to provide a stool sample by a nurse? by a doctor? by a receptionist? In some surgeries the receptionists give patients the stool collection pots, how do you feel about this? What did/*would* your family, partner, or friends’ think of you submitting a stool specimen? Is there anything that would make it difficult for you to return a stool sample to your practice? Is there anything that would make it easier for you to submit a stool sample to your practice? Why do you think some patients don’t return stool samples? How would you feel if whilst on a routine visit to your GP you were asked if you could collect a stool specimen for a study investigating different microbes in the gut? Once you had the stool in the pot, how easy did you find returning the sample to the surgery? What would you feel about being asked to post the sample back to the GP surgery if all of the packaging was provided? How do you think your GP practice could encourage people who take stool collection kits to return a sample?
**Part 3 – stool kits**
What would you expect to be in a stool collection kit? Information leaflets Can you look at these 2 information leaflets and discuss what you like/dislike about each Is there any other information you think should be included on the information leaflets? Do you think an information leaflet would be beneficial?

a Questions in italics were asked to participants selected who submitted a blood specimen, not a stool specimen, to the laboratory.
